# Mini-Review Regarding the Applicability of Genome Editing Techniques Developed for Studying Infertility

**DOI:** 10.3390/diagnostics11020246

**Published:** 2021-02-05

**Authors:** Bogdan Doroftei, Ovidiu-Dumitru Ilie, Maria Puiu, Alin Ciobica, Ciprian Ilea

**Affiliations:** 1 Faculty of Medicine, University of Medicine and Pharmacy “Grigore T. Popa”, University Street, no 16, 700115 Iasi, Romania; bogdandoroftei@gmail.com (B.D.); cilea1979@yahoo.com (C.I.); 2 Clinical Hospital of Obstetrics and Gynecology “Cuza Voda”, Cuza Voda Street, no 34, 700038 Iasi, Romania; 3 Origyn Fertility Center, Palace Street, no 3C, 700032 Iasi, Romania; 4 Department of Biology, Faculty of Biology, “Alexandru Ioan Cuza” University, Carol I Avenue, no 20A, 700505 Iasi, Romania; alin.ciobica@uaic.ro; 5 Department of Microscopic Morphology, Faculty of Medicine, University of Medicine and Pharmacy “Victor Babeș”, Eftimie Murgu Square, no 2, 300041 Timișoara, Romania; maria.puiu@gmail.com

**Keywords:** genome editing techniques, ZFN, TALEN, CRISPR-Cas9, infertility, zebrafish, mice, rats

## Abstract

Infertility is a highly debated topic today. It has been long hypothesized that infertility has an idiopathic cause, but recent studies demonstrated the existence of a genetic substrate. Fortunately, the methods of editing the human genome proven to be revolutionary. Following research conducted, we identified a total of 21 relevant studies; 14 were performed on mice, 5 on zebrafish and 2 on rats. We concluded that over forty-four genes in total are dispensable for fertility in both sexes without affecting host homeostasis. However, there are genes whose loss-of-function induces moderate to severe phenotypic changes in both sexes. There were situations in which the authors reported infertility, exhibited by the experimental model, or other pathologies such as cryptorchidism, cataracts, or reduced motor activity. Overall, zinc-finger nucleases (ZFNs), transcription activator-like effector nucleases (TALENs), and clustered regularly interspaced short palindromic repeat (CRISPR)/Cas9 are techniques that offer a wide range of possibilities for studying infertility, even to create mutant variants. It can be concluded that ZFNs, TALENs, and CRISPR/Cas9 are crucial tools in biomedical research.

## 1. Introduction

Concomitantly with provisions issued by the World Health Organization (WHO) and the English Oxford Dictionary, infertility can be defined as the impossibility to procreate, carry, or deliver a baby naturally after having been tried for at least one year [1]. However, there are disparities among definitions since WHO recommends at least two years of unprotected intercourse in order to validate these guidelines [1].

The origin of infertility in both sexes is complex and multifactorial [2]. Mutations are among the first driving forces that destabilize the human genome [3]. Even though the role of chromosomal aberrations in female infertility has already been well documented, that of monogenic mutations constituted the main objective of a recent review conducted by Guerri et al. [4]. The authors reported the role of forty-four genes: twenty-two are correlated to different pathologies such as primary ovarian failure (POF), ovarian dysgenesis (OD); seven concerning oocyte maturation defect (OOMD), preimplantation embryonic lethality (PREMBL), whereas five are attributed to recurrent pregnancy loss (RPRGL).

Papler et al. [5] aimed to assess the in vitro fertilization (IVF) parameters and gene expression of progesterone receptor (PGR) and tumor necrosis factor-inducible gene 14 protein (PTX3) in cumulus cells (CCs) of obese and normal weighting women. They did not find significant differences between obese and normal weighting women in terms of clinical pregnancies. The only necessary intervention was to increase the dose from 150 mcg to 300 mcg corifollitropin alfa and gonadotropin-releasing hormone agonist in obese women during in vitro fertilization (IVF) protocols. However, embryo quality on day five was poor. Moreover, they also noted an elevated expression of PGR and PTX3 in CCs from obese women.

There is an increasing interest in the current literature regarding how genotypes influence phenotypes [6]. Fortunately, genome editing methods arose in a fulminant manner and proved to be reliable for such interventions. This technology relies on sequence-specific nucleases (SSNs) to induce double-stranded breaks (DSBs) or single-stranded breaks (SSBS) at a targeted site within the genome. The process by which genome integrity is restored is ensured mainly through two major pathways known as nonhomologous final joint repair (NHEJ) and homology-directed repair (HDR) [7].

Zinc-finger nuclease (ZFN) [8] was the first engineered nuclease, followed in a relatively short interval by transcription activator-like effector nucleases (TALEN) [9] as a more flexible engineered nuclease. Both ZFN and TALEN consist of a sequence-specific DNA-binding module and a FokI nuclease domain. One specific feature of this restriction endonuclease is that it requires dimerization to become active. Exactly two modules are needed to be designed to target DNA sequence(s), allowing dimerization of FokI at the target DNA. ZFN and TALEN require a complex design for every different sequence target, and besides, there are several limitations in the creation of the full construct (plasmid and specific active nucleases), which renders these two editing tools quite expensive.

In contrast with ZFN and TALEN, clustered regularly interspaced short palindromic repeat (CRISPR)/Cas9 [10] remains the most powerful gene-editing tool since its emergence nine years ago. This system is based on two crucial components: Cas9 protein and guide RNA (gRNA) that form a complex. The gRNA is a small (~20 nucleotides) RNA which is complementary to the targeted sequence, being crucial for recruiting Cas9 protein to that site. CRISPR/Cas9 relies on the DNA-RNA interaction instead of DNA-protein, as is the case of ZFN and TALEN.

Thus, the present manuscript aims to highlight the main features and the associated results obtained through all these genome editing methods to study or even treating infertility in distinct experimental models.

## 2. Methodology

The databases used to extract data (until November 2020) were: ScienceDirect, PubMed/Medline, Scopus, and Cochrane Database of Systematic Reviews (CDSR). The searching strategy was: “ZFN and male infertility”, “ZFN and female infertility”, “zinc-finger nucleases and male infertility”, “zinc-finger nucleases and female infertility”, “TALEN and male infertility”, “TALEN and female infertility”, “transcription activator-like effector nucleases and male infertility”, “transcription activator-like effector nucleases and female infertility”, “CRISPR-Cas9 and male infertility”, “CRISPR-Cas9 and female infertility”, “clustered regularly interspaced short palindromic repeat and male infertility”, “clustered regularly interspaced short palindromic repeat and female infertility”, “genome editing methods and male infertility”, “genome editing methods and female infertility”, and “genome editing methods and infertility in experimental models”.

The adopted PubMed string was: (((genome editing technique[Title/Abstract] OR ZFN[Title/Abstract]) OR TALEN[Title/Abstract]) OR CRISPR-Cas9[Title/Abstract]) AND infertility[Title/Abstract]) AND experimental models[Title/Abstract] AND humans[Title/Abstract].

Three independent authors (B.D., O.-D.I., and M.P.) screened the titles and abstracts of the retrieved result; those that meet the eligibility criteria were further considered for a full-text review. Any discrepancy was solved by consent with a fourth author (C.I.).

We were unable to perform a formal quantitative meta-analysis due to the heterogeneity of data. Therefore, we employed the best evidence synthesis to identify the key results.

The main exclusion criteria were: (I) studies performed on other experimental models than zebrafish, mice, or rats; (II) or computational simulations. However, we will create a subsection dedicated to all studies that describe the applicability of combining two techniques and/or spermatogonial stem cells (SSCs).

We identified a total of 21 relevant articles that are chronologically summarized by the method in Table 1.

## 3. ZFNs and Infertility

ZFNs were developed twenty years ago [32,33]. ZFN constitutes the fusion of two artificial modular assemblies made up of three to six zinc-finger motifs found in proteins like transcriptional factors. The second constitutive component is the catalytic domain of a restriction enzyme known as FokI, naturally found in the bacterium *Flavobacterium okeanokoites* [34]. Based on the aforementioned, the whole concept behind ZFN was of great interest since its emergence. What defines ZFN is the high DNA-binding specificity of each zinc finger to recognize a combination of a maximum of three base pair sequence [8].

**Figure 1:**
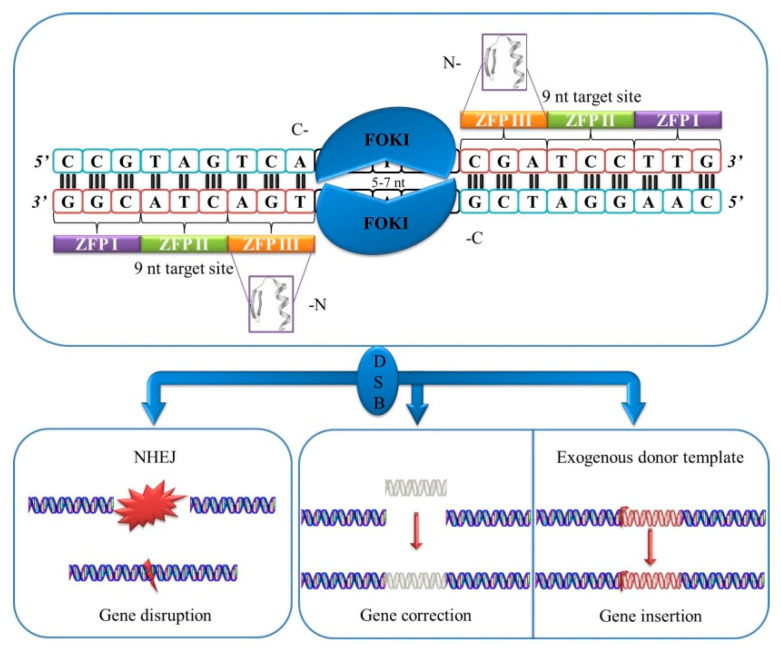
Schematic representation of zinc-finger nucleases (ZFNs).

ZFN induces targeted DSBs that subsequently are repaired through homology-directed repair (HDR). FokI cleaves when a dimer is formed [35]. Two monomers are required that attach to both forward and reverse strands of DNA to induce a DSB (Figure 1).

Aiming to investigate the role of disintegrin and metalloproteinase with thrombospondin motifs16 (Adamts16) in testes development, Abdul-Majeed et al. [11] used a ZFN-based gene-edited rat. It had been previously established by the same author that Adamts16 plays an important role in blood pressure. They showed that absence of Adamts16 lead to sterility and cryptorchidism in homozygotes. On the other hand, heterozygous male retained their normal phenotype. The testes of homozygous Adamts16-/- were smaller with no active spermatogenesis and associated with a gradual loss of the germ cells.

Rumi et al. [12] offered an insight into the pathophysiology of estrogen receptor gene knockout. They succeeded in generating Esr1-knockout rats by microinjection of the mRNAs encoding ZFNs that target the exon 3 of the Esr1 gene into single-cell rat embryos. They subsequently transferred these embryos to pseudopregnant recipients. Following this experiment, they observed five biallelic and one monoallelic mutant offspring. Though they attempted backcrossing with wild-type rats for all founders, fertility was evident only in the monoallelic Esr1 mutant female. By backcrossing the monoallelic model with a wild-type rat, the authors observed that offspring presented two specific deletions—482 bp (Δ482) or 223 bp (Δ223). Significant phenotypic alterations were observed in both specimens. More precisely, these deletions cause alternative splicing between exons 2 and 4, which renders a frameshift mutation and consequently the creation of premature stop codons just after Thr157. Female Δ482 was deprived of Esr1, thus explaining the lack of treatment efficiency with 17β-estradiol. Similar to Abdul-Majeed’s results, both sexes displayed particular features, while males showed small testes with enlarged dysplastic seminiferous tubules, in females, it was noted underdeveloped mammary glands, thread-like uteri, and pronounced polycystic ovaries.

It has been demonstrated that mutations in *RAB3GAP1* (RAB3 GTPase activating protein catalytic subunit 1), *RAB3GAP2* (RAB3 GTPase activating non-catalytic protein subunit 2), and *RAB18* (Ras-related protein Rab-18) cause Warburg micro syndrome 1–3 (WARBM1-3). Therefore, Park et al. [13] aimed to investigate the consequences of disturbing *TBC1D20* (TBC1 domain family member 20) functionality in mice since *TBC1D20* loss-of-function is related to WARBM4. *Blind sterile* (bs) mice carry a Tbc1d20-null mutation, displaying a distinct phenotype alongside *Rab3gap1^-/-^*, and *Rab18^-/-^).* Compared with experimental models, individuals affected by WARBM present indistinguishable clinical features. ZFN induced a *Tbc1d20* c.[418_426del] deletion that encodes a putative TBC1D20-ZFN with an in-frame p. [H140_Y143del] deletion within the TBC domain. Both *Tbc1d20^ZFN/ZFN^* and Tbc1d20^ZFN/bs^ strains exhibit cataracts, characterized by a thickened pupillary sphincter muscle and were infertile.

## 4. TALENs and Infertility

**Figure 2:**
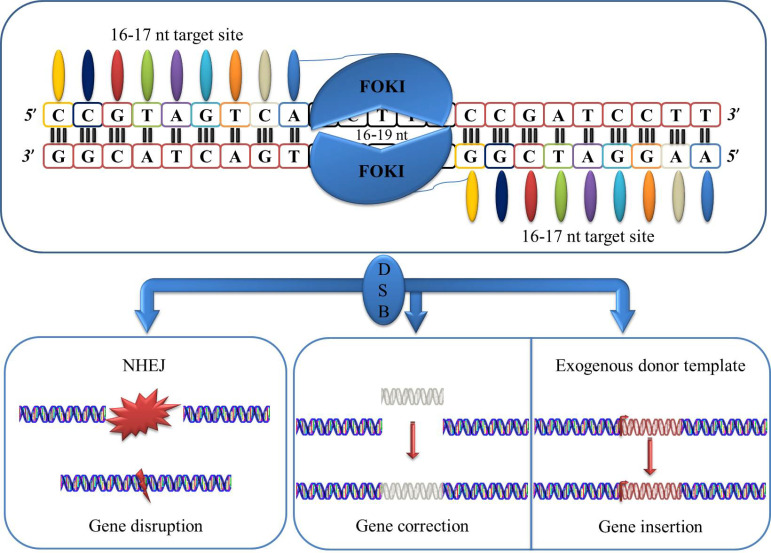
Schematic representation of transcription activator-like effector nucleases (TALENs).

Another set of DNA-binding proteins called transcription activator-like effectors (TALEs) was identified in *Xanthomonas* [36,37] soon after the emerge of ZFN. TALENs are artificial assemblies that consist of several TALE domain units (made up of 33–34 amino acids). The 12th and 13th residues are particular to each domain unit known as repeat variable diresidues (RVDs). Each recognizes specifically 1 bp of DNA. The most common RVDs for assembling TALE arrays are NI for adenine, HD for cytosine, NG for thymine, and NN or HN for guanine or adenine [38,39]. TALE DNA-binding domains can be constructed using numerous methods such as fast-ligation-based automatable solid-phase high-throughput (FLASH) assembly, iterative capped assembly, and ligation independent cloning [40]. This assembly is connected to the FokI endonuclease catalytic domain. Two TALE domains should be bound to DNA in order to FokI achieved to produce targeted DSBs that may be solved by HDR. The nonhomologous end-joining (NHEJ) mechanism by which these DSBs are frequently resolved is very useful to create KO models. TALENs offer two major improvements: (1) do not require selection or directed evolution and (2) improved specificity and reduced toxicity. However, one common attribute for both techniques is the induction of DSBs within interested DNA sequences [41,42] (Figure 2).

This has been discussed by Kato et al. [15,17] and further treated by Wang et al. [14] the crucial role of the Y chromosome in spermatogenesis, which have been created through TALEN rodent models with specific gene disruptions and insertions. Kato et al. [15] succeeded in inducing a sex reversal phenomenon in mice following TALEN RNA microinjection into pronuclear stage oocytes. They confirmed the presence of female external and internal genitalia. Female levels of blood testosterone and sexually dimorphic nucleus in the brain were noted. Nevertheless, subjects were infertile or had reduced fertility.

Wang et al. [14] had a similar approach, their main objective being to induce a mutation in EIF2S3Y. Similar to other studies, mice had a specific phenotype—hypoplastic testes and azoospermia. It can be concluded based on these studies that TALEN is a highly efficient method for generating experimental models of interest for studying sex chromosome Y. Mutagenesis can reach up to 35%, as has been recently shown. All seven mutant specimens were carriers of a mutation in forkhead box O3 (Foxo3(-/+), the five females were infertile in the first six months of life, whereas the two were males; one was subfertile, while the second one was fertile [18].

Knowing that histone variants alter chromatin biochemical makeup during development, including spermatogenesis, Anuar et al. [22] had as main objective to test the specificity of TALENs by disrupting H2A.B. The histone variant H2A.B (H2A.B.3) appeared late in evolution, involved in various processes, among which spermatogenesis is one of them. Anuar and co-authors used a single pair of TALENs to disrupt the H2A.B multi-copy gene family. Bioinformatics analyses based on Sanger and exome sequencing suggest no off-target mutation occurred following three consecutive generations. Male H2A.B.3 knockout mice were subfertile. The most noticeable characteristics were obstruction of the seminiferous tubules, abnormal sperm, and loss of protein complex Pol II.

Although zebrafish has become a widely used experimental model, we identified a limited number of reports. The deletion of hormone-specific β genes of both follicle-stimulating hormone (FSH) and luteinizing hormone (LH) in zebrafish led to numerous observations. The *fshb^-/-^* were fertile with a highly delayed development of genitalia. Despite having a normal gonadal growth, *lhb^-/-^* females were infertile. Thus, both hormones play a crucial role in female fertility status. While LH null mutant was infertile, null *fsh* leads to sexually reversed individuals. The authors concluded that neither *fshb* nor *lhb* mutation alone influences gonadal differentiation. Both sexes of fshb(-/-) were fertile, defined by significantly delayed testes and ovary development. Even though lhb(-/-) zebrafish exhibit normal gonadal growth, females were infertile. They also noted puberty onset delays in the fshb mutant following the use of previtellogenic follicles as a marker [16].

Tang et al. [21] and Xia et al. [20] deepen the spectrum in this case by establishing an androgen receptor (ar) and *Zmettl3^m/m^* zebrafish lines, respectively. Analogous to knockout homozygous mice, both sexes displayed specific phenotypes depending on the targeted gene. The levels of estradiol, 11-ketotestosterone (11-KT), and 17β-estradiol (E2) were significantly lower, this indicating defective steroidogenesis. The protein level of luteinizing hormone beta (LHβ) was also low by comparison with the wild-type strain. Overall, these findings indicate that such altered modifications have detrimental effects on reproduction for vertebrates.

Vanoevelen and co-authors moved research further by showing that galactose-1-phosphate uridylyl transferase (GALT) and N6-methyladenosine (m6A) could have clinical significance. Biochemical assays confirm that GALT activity is undetectable in knockout mutant homozygotes. Analogous to humans, fish exhibited an impaired motor activity and fertility prior to exogenous exposure to galactose. Even though classic galactosemia is a genetic disorder that apparently has no modulating influence on fertility, they demonstrated that *galt* knockout fish exhibited an impaired motor activity and fertility that mimics human pathophysiology [19].

## 5. CRISPR/Cas9 and Infertility

CRISPR/Cas9 is the latest system discovered in prokaryotes [43]. Bacteria and archaea adapted by developing a protective mechanism against the antagonistic action of phages and foreign plasmids. The corresponding CRISPR RNA (crRNA) is transcribed from the CRISPR locus in response to the entrance of a phage or foreign DNA. Its biological functionality and relevance depend on the ability to recognize and bind to foreign DNA together with an endogenous CRISPR- associated endonuclease (Cas9). DSB induces degradation of foreign DNA, and, in case this foreign DNA comprises a PAM recognition sequence, a foreign DNA fragment will be integrated at the 5’ end of the CRISPR locus.

The newly CRISPR/Cas9 system changed the entire perspective of editing the human genome. Cas9 endonuclease was also optimized to include nuclear localization signals to target DNA sequences of up to twenty base pairs [44]. For the scope of this review, we will, hereinafter, focus on its clinical applicability since all underlying concept of CRISPR-Cas9 has been extensively detailed by other scientists [45,46].

Tang, Kherraf and their collaborators enrolled over one hundred men diagnosed with multiple morphological anomalies of the sperm flagella (MMAF). The DNAH1, CFAP43, and CFAP44 orthologs [24,26] are the only known genes associated with MMAF that accounts for >40% of all cases. There were some controversies in twelve participants. Specifically, MMAF could not be genetically explained by any known mutation, but whole-exome sequencing (WES) revealed a 63% prevalence of mutations in autosomal candidate genes in homozygosity. Tang et al. [24] first generated two KO-deficient models in Cfap43 or Cfap44. Male mice were infertile, whereas the corresponding female mice were fertile. Kherraf et al. [26] deepen this spectrum one year later by creating four knockout (KO) and one knock-in (KI) lines through CRISPR/Cas9 to understand the physiopathology associated with these genetic variations. They generated four mouse lineages for the associated orthologs previously mentioned as well as the FlagC and FlagF genes. KI line was created by inserting the ssDNA template homologous to the targeted sequence together with the Cas9 construct plasmid. The HA tag added to the ssDNA is merely used to facilitate the detection in vivo of the targeted protein. Approximately 31% of the live pups suffered a mutational event—indels near the PAM sequence, while in 30% of the cases was observed a high rate of germline mosaicism of the F1. The biallelic mutations in CFAP43 or CFAP44 could cause an impairment of sperm motility and flagellar abnormalities. It was concluded that mutational events induced by CRISPR/Cas9 persisted in several cell divisions following the injection.

Abbasi et al. [30] targeted three distinct genes that apparently are involved in cell differentiation and linked to the female reproductive system. They discover that triple KO mutant mice (TKO) for *Oosp* family genes are not infertile, rather defined by a reduced prolificacy. Unfortunately, there are many gaps in our knowledge. Zinc finger Y-chromosomal protein (Zfy) was first proposed to be a sex determination factor, but its biological function has just only been recently elucidated. Nakasuji et al. [47] revealed that sole mutations in Zfy1 and Zfy2 do not promote phenotypical changes in mice. However, double knockout models were infertile and exhibited numerous structural abnormalities.

The less-documented yet crucial are ubiquitin-specific peptidase 26 (USP26) and PARN-like ribonuclease domain containing 1 (PNLDC1). Analogous to most genes reported, these two are also positively associated with sperm defects in males [48,49]. Usp26 mutant males backcrossed with DBA/2 were sterile or subfertile and had atrophic testes. Similar clinical presentations were noted in mice following a mutation in Pnldc1. Mutations in Pnldc1 inhibit piwi-interacting RNA trimming that may culminate in a reduction of mature piwi-interacting RNAs in the testis.

Singh and Schimenti [23] investigate the segregation phenomenon through CRISPR/cas9 by creating putatively deleterious nonsynonymous SNPs (nsSNPs) in the mouse orthologs of fertility genes. They discovered that of these four mutated essential meiosis genes, only a cyclin-dependent kinase 2 (Cdk2) allele mimicking SNP rs3087335, which alters an inhibitory WEE1 protein kinase phosphorylation site, caused infertility.

It is well-known that Fanconi anemia (FA) is positively correlated with developmental disturbances. Ramanagoudr-Bhojappa and his collaborators [27] demonstrated that of all loss-of-function mutants created for seventeen FA genes as well as two genes encoding FA-associated proteins, only the *fancd1* and *fancj* knockout males showed infertility. To conduct this experiment, they elected two indel mutations known to induce premature truncations within all panel of genes targeted and further confirmed through RT–PCR. In total, were generated 36 lines from 19 genes, with the mention that 4 indels promoted defective splicing. They subsequently performed a series of analyses to confirm these findings, only *fancp-/-* fish displaying gross developmental disturbances.

Tang et al. [28] offered an in-depth view regarding the applicability of CRISPR-Cas9. This system was used to induce indels at the boundary of exon and intron; it caused alternative splicing at the 5′ splice site that led to multiple different mRNAs. Accordingly, promoted a premature termination codon characterized by a downregulation of the gene.

Among all one thousand genes assumed to have a role in fertility and hypotheses issued, partially proved to be dispensable since over forty-five genes knockout did not affect the host’s homeostasis [29,30,31]. For example, spermatogenesis associated 16 (Spata16), actin-like 7A (ACTL7A), or Zfy1/2-DKO mice are among the many others that are crucial for fertility. Loos-of-function cause spermatogenic or early embryonic arrest/failure. Recently, was observed a reduced expression and abnormal localization of phospholipase C zeta (PLCζ) in both ACTL7A/Actl7a-mutated men and mice [25,47,50].

Considering the lack of male contraceptives compared with women, searching for new candidate proteins for non-hormonal contraceptives is essential. Park et al. [51] applied in silico approaches and identified 10 testis-abundant genes in humans and mice that are dispensable for fertility. Following the investigation made, they concluded that neither of these 10 genes nor 4930402F06Rik and 4930568D16Rik genes, which are 3 Glt6D1 paralogs found in mice but not in human, are relevant for male fertility.

## 6. Alternative View

We will begin this section by summarizing in Table 2 all studies found in the current literature regarding SSCs and a combination of known editing methods. As mentioned above, we will not discuss those articles in which experimental models other than mice, rats, and zebrafish were used. From all studies presented in Table 2, we will focus only on three of them.

**Figure 3:**
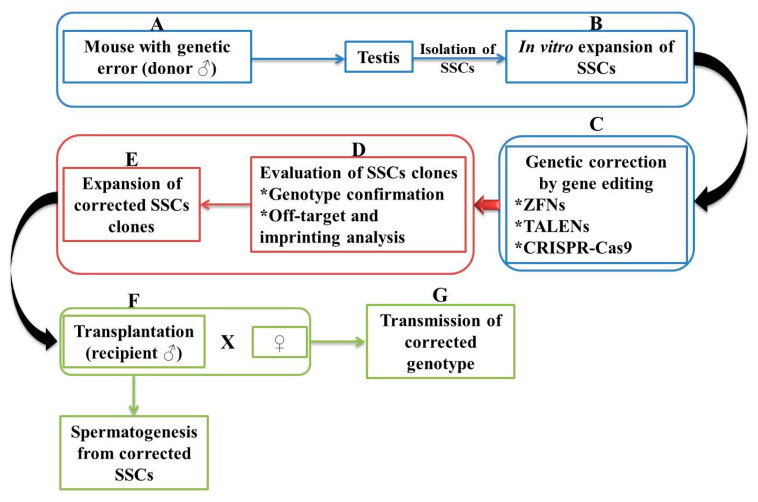
Schematic representation of germline gene therapy by spermatogonial stem cells (SSCs). SSCs are first isolated from the testis of a mouse with a genetic error (**A**). SSCs are expanded in culture (**B**) and the genetic error-corrected through one method (ZFN, TALEN, or CRISPR-Cas9) (**C**). To confirm that is no genetic error, SSCs are analyzed (**D**) and again expanded in the culture being obtained corrected clones (**E**). SSCs are transplanted into an infertile recipient, a process in which spermatozoa are produced (**F**). Progeny is generated by pairing with a wild-type female or micro insemination into the oocyte of the wild-type strain (**G**). The asterisk indicates a numbering.

Spermatogonial stem cell transplantation (SSCT) is viewed as a biomedical research tool attributed to the transfer of germline between a donor and a recipient (Figure 3). Sato et al. [57] tested the specificity of TALEN and double-nicking CRISPR/Cas9 on SSCs by targeting Rosa26 and Stra8 loci. The experiments were carried out starting from the hypothesis according to which both loci are dispensable and indispensable in spermatogenesis, respectively. Harvested SSCs cell colonies manifested a high targeting efficiency, Rosa26-targeted cells differentiated into fertility-competent sperm following transplantation. It has been shown that Stra8-targeted GS germline stem cells showed impaired spermatogenesis after transplantation. This further suggests the fundamental role it plays in initiating meiosis.

Nonobstructive azoospermia (NOA) is defined as one of the most severe forms of male infertility. Considering that NOA is characterized by the absence of sperm after ejaculation due to failure of spermatogenesis, Li et al. [58] aimed to restore fertility in mouse heterozygous mutant for a c-Kit gene (Kit^w^/Kit^wv^). They first isolated mutant SSCs from one single unilateral testis of a two-week-old specimen propagated in vitro. These mutant SSCs were defined by a point C to T mutation on the Kit^wv^ site that was corrected through CRISPR-Cas9-mediated homology-directed repair (HDR) in vitro. The resultant SCCs were subjected to a series of tests and transferred into the remaining testis, being accomplished spermatogenesis into the recipient. Four months after transplantation, the authors obtained, through this approach, healthy offspring, carriers of either wild-type *c-Kit* gene or Kit^w^ mutation. Ciccarellia et al. [59] deepened this sphere and demonstrated that all these mammals had structurally normal testes but were germline ablated. On the other hand, prepubertal allogeneic SSCT mice were naturally fertile.

## 7. Conclusions

Based on all aspects discussed throughout this manuscript, the following conclusion can be drawn. FZN, TALEN, and CRISPR-Cas9 are useful for exploring, deciphering, and studying infertility regardless of sex. Although CRISPR-Cas9 has received more attention and interest from researchers, potent results were obtained with the three genome-editing tools. Although not as widely used by comparison with CRISPR/Cas9, both ZFN and TALEN possess high specificity for creating different specific models. This argument is supported by the fact that all these three genome editing tools have been successfully applied to treat other conditions. This mini-review offers a conclusive perspective regarding all studies performed until the present.

## Figures and Tables

**Table 1:** Summary content regarding the applicability of genome editing methods for studying infertility.

Method	Experimental Model	Gene(s) Targeted	Phenotype Induced	Year of Publication	Reference
ZFN	Rats (injection)	ADAMTS-16	Bilateral cryptorchidism and infertility	2014	[11]
	Rats (microinjection)	Esr-1	Small testes, epididymides, and seminal vesicles Large polycystic ovaries devoid of corpora lutea and narrow, thread-like uteri	2014	[12]
	Mice (injection)	Tbc1d20	Vacuolated cataracts and testicular abnormalities	2014	[13]
TALEN	Mice (injection)	Sry, Uty	Anatomical sex reversal	2013	[14]
	Mice (microinjection)	Sry	Anatomical sex reversal	2013	[15]
	Zebrafish (injection)	Deletion of hormone- specific β-genes of both FSH and LH	(Fertile)—significant delay of ovaries and testes development in both FSH-deficient zebrafish (Infertile)—normal gonadal growth, but failed to spawn in LF-deficient zebrafish	2014	[16]
	Mice (microinjection)	Eif2s3y	Azoospermia	2015	[17]
	Mice (injection)	Foxo3	Five infertile female mouse One subfertile and one infertile male mouse	2015	[18]
	Zebrafish (injection)	GALT	Reduced motor activity and impaired fertility	2017	[19]
	Zebrafish (injection)	mettl3	Significant lower FG and hCG both in vitro and in vivo Reduced sperm maturation and motility Significantly reduced levels of 11-KT and E2 levels and defective gamete maturation	2018	[20]
	Zebrafish (microinjection)	ar	Small testes and decreased levels of 11-KT, estradiol, and LHβ	2018	[21]
	Mice (injection)	H2A.B, H2A.B.3	Subfertile specimens displaying abnormal sperm and clogged seminiferous tubules	2019	[22]
CRISPR-Cas9	Mice (microinjection)	Cdk2	Infertility and germ cell-depletion in homozygous	2015	[23]
	Mice (injection)	Cfap43, Cfap44	MMAF phenotype in male mice, whereas the female mice were fertile	2017	[24]
	Mice (injection)	SPATA16	Point mutation was not essential, but deletion of the 4^th^ exon of Spata16 led to a spermatogenic arrest, not globozoospermia	2017	[25]
	Mice (injection/ electroporation)	Cfap43, Cfap44, FlagC, FlagF	Live pups displayed either insertions or deletions in one of the targeted regions, and up to 30% of mosaicism	2018	[26]
	Zebrafish (microinjection)	fanca, fancb, fancc, fancd1/brca2,fancd2, fance, fancf, fancg, fanci, fancj/brip1, fancl, fancm, fancn/palb2, fanco/rad51c, fancp/slx4, fancq/ercc4, fanct/ube2t, faap100 and faap24	Partial or complete sex reversal from female-to-male All male and female models were fertile with the exception of two specimens that had mutations in fancd1 and fancj	2018	[27]
	Mice (microinjection)	Ccnb3	Downregulation of Ccnb3 did not affect the knockout model during embryo development, spermatogenesis, and fertility status	2018	[28]
	Mice (microinjection/ electroporation)	1700001O22Rik, 1700010B08Rik, Ankrd7, Banf2, Bpifa3, Cct6b, Fam221b, Fndc8, Gsg1, Hmgb4, Hyal6, Mgat4d, Morn3, Oxct2a, Oxct2b, Scp2d1, Slc36a3, Tex13a, Tex13b, Tex35, Tgif2lx1, Tktl2, Tmem202, Tmem270, Trim17, Trpd52l3, Ube2d2b, Ubqln5, Usp26, Vwa3b	Normal fecundity	2019	[29]
	Mice (injection/ electroporation)	Oosp1,Oosp2, Oosp3, Cd160, Egfl6	Fertile, but exhibited a decreased prolificacy	2020	[30]
	Mice (microinjection/ electroporation)	4921507P07Rik, Allc, Cabs1, Fam229b, Fscb, Hdgfl1, Iqca, Lelp1, Spata24, Tmem97, Eddm3b, Lrcol1, Tmem114	Fertile and without any phenotypic change(s)	2020	[31]

FSH—follicle-stimulating hormone; LH—luteinizing hormone; FG—full-grown; hCG—human chorionic gonadotropin; 11-KT—11-Ketotestosterone; E2—estradiol; LHβ—luteinizing hormone beta polypeptide; MMAF—multiple morphological anomalies of the sperm flagella.

**Table 2:** Overview regarding the reliability of genome editing methods by using non-conventional experimental models.

Method	Experimental Model	Year of Publication	Reference
CRISPR-Cas9 and TALEN	Pig, goat and cattle	2013	[52]
TALEN	Bombyx mori	2014	[53]
TALEN	Bombyx mori	2020	[54]
TALEN	Bombyx mori	2019	[55]
TALEN	Drosophila melanogaster	2015	[56]
CRISPR-Cas9 and TALEN—SSCs	Mice	2015	[57]
CRISPR-Cas9—SSCs	Mice	2019	[58]
CRISPR-Cas9	Mice, pigs, goats and cattle	2020	[59]
